# The composition of contemporary American and Swedish smokeless tobacco products

**DOI:** 10.1186/s13065-019-0548-0

**Published:** 2019-03-19

**Authors:** Kevin G. McAdam, Harriet Kimpton, Arif Faizi, Andrew Porter, Brad Rodu

**Affiliations:** 1 British American Tobacco, Group Research and Development, Regents Park Road, Southampton, SO15 8TL UK; 2 3810 St. Antoine W, Montreal, QC H4C 1B4 Canada; 3 University of Louisville, Clinical Translational Research Building, 505 South Hancock Street, Louisville, KY 40202 USA

**Keywords:** Smokeless tobacco, Snus, Snuff, Water measurement, Moisture content

## Abstract

The major components of 70 brands of smokeless tobacco products (STPs) from Sweden and the US were determined to provide greater understanding of the general chemical composition of these products. Various styles of STPs were examined: loose and portion snus from Sweden, and chewing tobacco, dry snuff, moist snuff, hard pellet, soft pellet and plug from the US. The components analysed were major STP components such as water, nicotine, sugars, humectants, sodium ions, chloride ions and ash. The relative quantities of the components varied significantly between different styles of STP. The major component of moist snuff and Swedish loose snus is water. With Swedish portion snus water and pouch material comprise more than half of the product mass; with chewing tobaccos water and sugars comprise around 60% of the products. With these STPs, tobacco was a minor component (30–35%) of the product mass. By way of contrast, tobacco comprised the majority (around 70–90%) of the product mass with dry snuff, hard pellet and soft pellet products. Additives such as sugars, propylene glycol, glycerol, and sodium chloride comprised up to around 12% of the STPs, except for plug and chewing tobaccos where sugars comprised 15–30% by mass of the STP on average. Significant disagreements were found amongst alternative methods of determining water/moisture content for STPs. In particular the oven method, commonly used to determine moisture in tobacco, gave significantly higher values than the Karl Fischer water method when propylene glycol was present. Smaller but similar differences were found using the Near-Infrared method. Choice of measurement technique has important consequences for accuracy of toxicant levels when reporting on a dry-weight basis, a commonly used parameter in smokeless tobacco research and emerging regulatory standards. Conversion to a DWB was also found to produce a preferential bias between and within different STP categories in favour of drier products. These data provide greater understanding of differences in the compositions of contemporary smokeless tobacco products, and demonstrate challenges associated with conversion of actual product contents to dry weight basis values.

**Electronic supplementary material:**

The online version of this article (10.1186/s13065-019-0548-0) contains supplementary material, which is available to authorized users.

## Introduction

Although Smokeless Tobacco Products (STPs) have been designated as Group 1 carcinogens i.e. carcinogenic to humans [1, 2], there is growing acceptance that different product styles can offer different levels of health risk in line with their toxicant contents [3]. This has prompted a series of analyses of different STP styles for a wide range of toxicants and carcinogens, including tobacco specific nitrosamines (TSNA) [1], metals [4, 5], volatile aldehydes [6], polycyclic aromatic hydrocarbons (PAH) [7, 8], hydrazine [9], acrylamide [10], radioisotopes [11], ethyl carbamate [12] and coumarin and angelica lactones [13].

Concurrent with publication of these analyses has been the introduction of regulations focusing on reporting levels of various components of tobacco products (including STPs). Historically these have included Federal rules published by the Center for Disease Control [14] for nicotine, regulations by the State of Massachusetts [15] to report nicotine contents and by the State of Minnesota [16] to identify the presence of detectable levels of ammonia (or ammonia compounds), arsenic, cadmium, formaldehyde and lead. The State of Texas [17] required disclosure of product ingredient information including nicotine content, and the State of Utah [18] required disclosure of the moisture content of STPs. These reporting requirements for STPs were complemented by the introduction of FDA oversight of tobacco products that began in 2009 following the introduction of the Family Smoking Prevention and Tobacco Control Act. [19]. Through its Tobacco Product Scientific Advisory Committee (TPSAC) the FDA initially assembled a list (“The Established List”) of 93 “Harmful or potentially harmful constituents” (HPHC) of tobacco products including 79 that are designated as carcinogenic as well as constituents that are respiratory toxicants, cardiovascular toxicants, reproductive toxicants or addictive. Recognising the existence of time and resource constraints in 2012 the FDA required only an abbreviated set of constituents to be reported; for STPs nine constituents were selected: acetaldehyde, arsenic, benzo[a]pyrene (B[a]P), cadmium, crotonaldehyde, formaldehyde, nicotine (total and free), 4-(methylnitrosamino)-1-(3-pyridyl)-1-butanone (NNK) and N’-nitrosonornicotine (NNN) [20]. Of these, arsenic, B[a]P, cadmium, formaldehyde, NNK and NNN are Group 1 carcinogens [2]. The FDA requires toxic constituents to be reported either by portion (where appropriate) or by weight of material on an “as sold” i.e. wet weight basis (WWB).

There have also been proposals to regulate toxicant contents of STPs. The World Health Organisation (WHO) Tobacco Product Regulation (TobReg) study group [21, 22] have presented proposals to limit toxicant contents of STPs. The emphasis of the WHO regulatory proposals was to set upper limits of 2 μg/g dry weight of tobacco for the combined concentrations of the TSNAs NNN and NNK, and 5 ng/g dry weight of tobacco for B[a]P. The proposal was identified as a product standard rather than a measure of human exposure. Dry weight basis was selected as the metric for recommendation by WHO, largely on the basis that it is in accordance with established historic laboratory practice and has long term use as a method of standardising measurements of STP constituents. It operates by adjusting for differences in moisture/water content amongst products. The WHO did not identify any preferred methods for the measurement of moisture or water necessary to convert actual product levels to dry weight data. In 2017 the FDA announced plans for a  standard concerning the NNN content of STPs [23]. The proposed limit was 1 μg/g dry weight NNN; conversion of wet weight measured NNN STP concentrations to dry weight values was proposed to be determined according to International Organization for Standardization (ISO) standards ISO 6488:2004 [24] and ISO 6488:2004/Cor 1:2008 [25] (Karl Fischer measurement) or ISO 16632:2013 (gas chromatographic measurement of water) [26].

Dry weight basis measurements are surprisingly challenging to conduct with acceptable accuracy and precision due to a lack of consistency in the measures used to convert from actual product contents to dry weight values. Two concepts have been used to convert from wet weight to dry weight values. The first is water content, and the second is moisture content. Moisture content is a broader principle than water content, as it is not exclusive to water, and moisture contents are heavily influenced by the presence of other volatile compounds. Various methods of measuring moisture or water in tobacco are in use, and CORESTA has summarised the methods and their strengths and weaknesses [27]. Methods for moisture determination include thermal oven methods, and microwave oven methods [28]. Methods for water determination include several variations of the Karl Fischer method [29], azeotropic distillation with benzene or cyclohexane [30], near infra-red (NIR) spectroscopy [31] and gas chromatography [32]. CORESTA initially developed two methods that were subsequently further developed into ISO-certified standards for measuring water in tobacco and tobacco products in the range 2–55%. One uses the Karl Fischer method with potentiometric titration [24, 25, 33] and the other uses gas chromatography [26, 32]. Interlaboratory testing conducted by CORESTA using dry and moist snuff products as well as standard and cigarette tobaccos found that both gave equivalent results. However, comparison of the methods with Indian STPs showed higher values from the gas chromatographic method, due to its lack of chemical specificity [34], suggesting that further evaluation of water determination methods with a broader range of STPs is warranted.

Surprisingly, given the considerable regulatory focus on STP toxicant contents, an area that has received relatively little attention to date is the general composition of STPs. The general production and manufacture of STPs is reasonably well characterised, [1, 35], and it is widely known that contemporary STPs contain additives. For example, Going et al. [36] and Hsu et al. [37] measured sugars in US products and deduced that sugars were added to some styles of STPs. Foulds et al. [38] noted that Swedish snus contains 45–60% water, 1.5–3.5% sodium chloride, 1.5–3.5% humectants (e.g. propylene glycol and glycerol), 1.2–3.5% sodium bicarbonate, and up to 1% flavouring. Similarly, Swedish Match, a major manufacturer of Swedish snus, has published a detailed list of all additives and their levels by brand in both loose and portion snus products. Swedish Match products contain water, propylene glycol and/or glycerol, sodium chloride, sodium carbonate and flavours [39]. Moreover, portion snus products are wrapped in a paper-like fleece material. However, to date there has not been a systematic study comparing additive levels and major constituents of all contemporary STPs in a consistent manner. The aim of the present study was therefore to fill this gap in understanding of STP compositions and also, given the importance of water/moisture measurement in converting actual toxicant contents to dry weight basis values, to examine the suitability of different analytical methods for these parameters. In our study we used the Karl Fischer method as the reference point and compared three commonly used water and moisture determination methods across a range of contemporary Swedish and US STPs.

## Methods

### Products

The products which were used to generate the data for this report have already been described [8]. In total there were 70 STPs obtained from the US and Sweden in 2008–2009. These consisted of 5 dry snuffs (DS), 16 moist snuffs (MS), 13 chewing tobaccos (CT), 2 hard pellets (HP), 1 soft pellet (SP) and 1 plug from the US and 10 loose snus (L Snus) and 22 portion snus (P Snus) from Sweden. P Snus (mini or normal size) is pre-packaged tobacco powder in small porous bags termed “pouches”. We also sampled 66 of these STPs in 2010 to examine the effect of different approaches for moisture and water determination on conversion of wet weight data to dry weight values.

The Swedish products were sourced from Swedish retail websites, imported into the United Kingdom, and kept frozen at − 20 °C until analysis. The products represented seven different manufacturers and accounted for ca. 89% of the market share of STPs in Sweden in 2008. The American products were sourced from shops in the United States, imported, and kept frozen at − 20 °C until analysis. The products represented 9 different manufacturers and accounted for ca. 88% of the market share for the major STP categories in 2008.

These 70 STPs were analysed at British American Tobacco (BAT) for contents of major constituents previously reported to be present in STP including nicotine, total and reducing sugars, propylene glycol, glycerol, sodium ions, chloride ions, ash and oven moisture. Moisture analyses were repeated using an alternative oven method (at Labstat International, Kitchener Ontario), and two methods were also used to determine water contents, near infra-red (NIR) spectroscopy and the Karl Fischer method. The pouch and tobacco weights for the P Snus products and the pellet weights for the SP and HP products were also determined.

### Analysis methods

Analysis methods for some of the analytes presented in this study have been reported previously. For example, the method for Karl Fischer water analysis was reported by McAdam et al. [9]. Methods for nicotine, pH, reducing and total sugars, glycerol and propylene glycol, sodium and chloride ions were described by McAdam et al. [12]. NIR water and ash content methods were reported by McAdam et al. [11]. Individual methods are summarised below.

### Masses of portion products

Pellets. Pellet products were weighed directly. Tobacco was removed from the cellulose pouches of 12 frozen P Snus samples and the average weights of tobacco and pouch were determined.

### Oven moisture analysis

Oven determinations of STP moistures were conducted in two laboratories, Labstat International (Kitchener Ontario), and at BAT's Southampton UK laboratories. Labstat followed AOAC Method 966.02 [40] while BAT measurements were conducted using a modification of this method with the oven temperature at 110 °C rather than the 99.5 ± 0.5 °C specified by AOAC.

### Water content by Karl Fischer Analysis

The Karl Fischer analysis method for tobacco water was conducted using the method described in ISO 6488:2004 [24, 25], adapted slightly to smaller sample sizes and volumes so as to be compatible with the KEM MKC-500 analyser (Kyoto Electronics, Tokyo, Japan) used for these measurements. Instead of using 5 g of sample, extraction in 50 ml anhydrous methanol for 30 min, and titration of 10 ml of the extract, in this study we used 2 g STP to which 20 ml anhydrous methanol was added and the sample sonicated for 15 min before standing for at least 2 h to ensure complete extraction. Large tobacco particle size samples (such as soft pellet) were reduced to less than 4 mm to aid extraction. 100 μl of methanol was sampled and injected into the Karl Fischer analysis cell. Water blanks were subtracted, and analyses conducted in triplicate.

### Water content by NIR spectroscopy

The water content of all STPs was measured by near-infrared (NIR) spectroscopy using a standard technique wherein water was extracted from the STPs using anhydrous methanol. A calibrated double-beam spectrometer was used to measure the intensity of the combination band at 1943 nm (due to –OH stretching and H–OH bending of the water molecule); intensities were compared to standards containing water in methanol for the purposes of quantification.

### Ash content

The ash content of STPs was estimated by heating the STP in air in a muffle furnace at 500–550 °C in a pre-dried silica dish for 1 h. Organic material present in the sample during this time period was burnt off as combustion gases; if the resulting ash was not uniformly white (the presence of dark colour in the ash indicates incomplete ashing) then the samples were heated for a further 30 min. The sample weight after ashing, allowing for the STP’s original moisture content, allowed calculation of the STP’s inorganic content.

## Results and discussion

Mean values for some of the data presented in this study have been reported previously, and some new data are also presented. The sources of the data are as follows:

For the Swedish products previously published data on sodium and chloride ion contents and pH values [12], and ash contents [11] are repeated here together with new information in the form of analytical variability (standard deviations). In addition, new data are presented here for mean and SD values for reducing and total sugars, glycerol and propylene glycol contents of these products, as well as pouch weights of the P snus products.

For the US products, with MS, Plug, CT, HP and SP products new data are presented here for nicotine contents; the other content data have been published previously [11, 12]; although analytical variability data for these previously reported measurements are presented here for the first time. With DS products new data are presented for nicotine, and for reducing and total sugars; other data for DS have been reported previously [11, 12].

In respect of water and moisture analysis data for all products, previous data for Karl Fischer water [9] and NIR water contents [11] are combined with new data for Oven moisture measurements.

### Major components of STPs

Data on the contents of major, non-toxicant components of the 70 STPs measured in this study are summarised in Table 1 and tabulated in detail in Additional file 1: Tables S1–S8. These tables show the average concentrations of total and reducing sugars, humectants (propylene glycol and glycerol), sodium ions, chloride ions, ash, nicotine on an “as sold” (i.e. WWB) basis, together with pH values for these STPs. Some of the components, such as nicotine are naturally present in tobacco and some such as propylene glycol are added during manufacture. Others, such as glycerol, sugars and sodium and chloride salts, may have contributions from both the tobacco and from additives.

**Table 1:** Summary of components and pH for STPs examined in this study on a wet weight basis

STP	Portion snus	Loose snus	Chewing tobacco	Moist snuff	Dry snuff	Plug	Hard pellet	Soft pellet
N	22	10	13	16	5	1	2	1
Reducing sugars (%)								
Mean	0.70	0.73	13.7	0.17	0.56	12	4.35	2.1
SD	0.17	0.22	6.60	0.08	0.48	–	1.06	0
Total sugars (%)								
Mean	0.63	0.70	31.6	0.13	0.5	14.9	5.05	5.4
SD	0.20	0.19	6.10	0.04	0.62	–	0.92	0
Ash (%)								
Mean	16.3	13.6	11.0	16.7	21.5	12.8	21.7	13.3
SD	3.20	0.95	1.66	0.82	1.65	0.18	1.35	0.02
Glycerol (%)								
Mean	0.05	3.43	3.1	0.47	0.09	1.69	0	0.11
SD	0.04	1.37	1.22	1.35	0.08	0.12	0	0
Propylene Glycol (%)								
Mean	2.93	3.02	0.4	0.00	0.008	0.62	0	0
SD	1.23	0.66	0.25	0.01	0.02	0.01	0	0
Na^+^ (%)								
Mean	2.56	2.32	0.7	3.15	0.274	1.46	0.04	0.17
SD	0.64	0.32	0.08	0.37	0.40	0.03	0	0
Cl^−^ (%)								
Mean	3.63	3.53	1.4	5.41	1.31	2.45	0.37	0.27
SD	1.24	0.60	0.17	0.35	0.36	0.03	0.04	0.01
Nicotine (mg/g)								
Mean	9.87	7.93	6.7	12.2	20.6	10.9	8.4	29.5
SD	4.14	1.18	1.84	0.08	3.08	–	2.26	–
pH								
Mean	8.46	8.49	6.1	7.8	6.4	5.3	8.0	5.3
SD	0.41	0.39	0.27	0.08	0.51	0.13	0.18	0.0

**Fig. 1:**
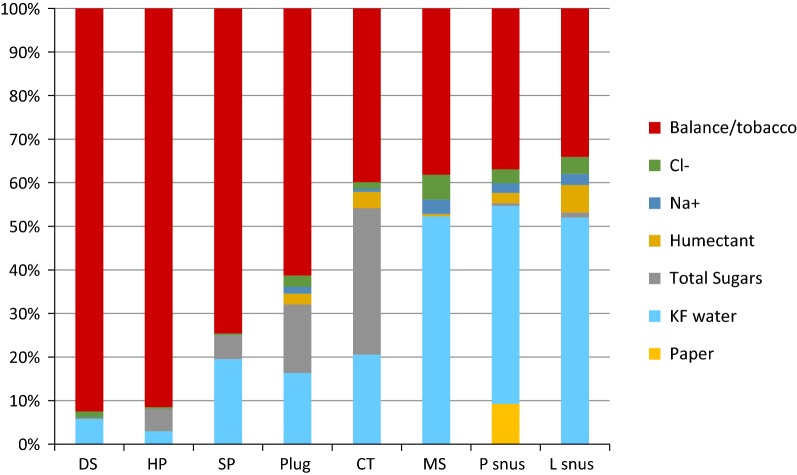
Average concentrations (%) of the major components of STPs by product style

Table 1 shows that there are distinct differences in composition across the various styles of STP. This is also illustrated in Fig. 1, which shows the average percentages of water content, total sugars, glycerol, propylene glycol, sodium ions, and chloride ions for each style of STP as a stacked bar chart. The “balance” is the amount by which the sum of the measured components differs from 100%. It can be assumed that most of the “balance” consists of tobacco, although the presence of relatively high levels of inorganic species in Ariva hard pellet has been noted previously [11], and the use of STP flavourings will also impact on this value. In the case of P Snus the weights of fleece comprising the pouch have also been included (Table 2). The proportions of fleece in these products ranged from 6.5 to 15% with an average of 9.6%.

**Table 2:** Portion, tobacco and paper weights for portion STPs

Smokeless products		Portion	Component weights (% of portion)	
		Weights	(g)	
		(g)	Tobacco	Paper
General white	P snus	0.878	0.811 (92.4)	0.067 (7.6)
General, mini		0.452	0.388 (85.8)	0.064 (14.2)
General		0.926	0.842 (90.9)	0.084 (9.1)
Ettan		0.822	0.744 (90.5)	0.078 (9.5)
Grovsnus		0.931	0.845 (90.8)	0.086 (9.2)
Grovsnus White		0.846	0.781 (92.3)	0.065 (7.7)
Goteborgs Rape White		0.906	0.837 (92.4)	0.069 (7.6)
Kronan		0.887	0.794 (89.5)	0.093 (10.5)
Catch Licorice, mini		0.44	0.382 (86.8)	0.058 (13.2)
Catch White Licorice		1.042	0.969 (93.0)	0.073 (7.0)
Catch Dry White Eucalyptus, mini		0.331	0.286 (86.4)	0.045 (13.6)
Catch Dry White Licorice, mini		0.32	0.272 (85.0)	0.048 (15.0)
Granit White		0.805	0.748 (92.9)	0.057 (7.1)
Granit		n/a		
Tre Ankare White		0.89	0.825 (92.7)	0.065 (7.3)
Level		n/a		
Skruf strong		0.983	0.903 (91.9)	0.08 (8.1)
LD original		0.893	0.802 (89.8)	0.091 (10.2)
Knox		0.867	0.794 (91.6)	0.073 (8.4)
Wise Citrus and Menthol (6 mg)		0.275	0.257 (93.5)	0.018 (6.5)
Romeo y Julieta		0.993	0.893 (89.9)	0.1 (10.1)
1847 original		0.889	0.807 (90.8)	0.082 (9.2)

#### Water/moisture contents

**Fig. 2:**
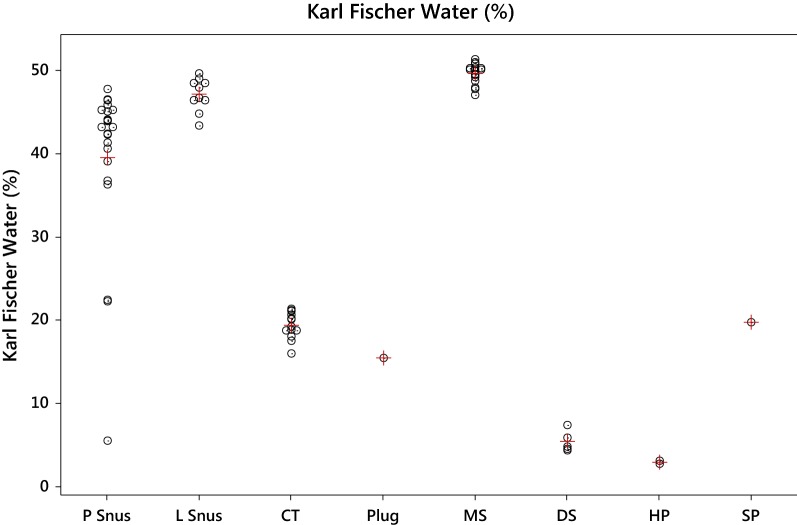
Individual (o) and average (+) values for Karl Fischer water content (%) by product style

In this study moisture and water contents of the STPs were determined by several different methods, the results of which will be discussed in a later section. Using the Karl Fischer water results (Tables 3, 4 and 5), average water contents across different STP styles were calculated as follows: MS (49.6%) > L Snus (47.1%) > PSnus (39.5%) > SP (19.7%) > CT (19.3%) > DS (5.6%) > HP (2.9%). As shown in Fig. 2, within each style of STP the water contents for individual products were similar to each other except for the P Snus category where three of the products had significantly lower water contents than the average: Catch Dry White Eucalyptus Mini (22.4%), Catch Dry White Licorice Mini (22.2%) and Wise Citrus and Menthol (5.5%).

**Table 3:** Water and moisture contents for Swedish STPs

	Style	Water (%)		Moisture (%)		BAT oven-KF (%)	Labstat oven—KF (%)	NIR—KF (%)
		Karl Fischer	NIR	Labstat oven	BAT oven moisture			
General White	P snus	45.3	48.7	53.5	55	9.7	8.2	3.4
General mini		44.1	46.5	47.4	52.2	8.1	3.3	2.4
General		41.3	48.6	50.6	54.8	13.5	9.3	7.3
Ettan		42.3	46.4	49.2	52.3	10	6.9	4.1
Grovsnus		43.9	45.5	49.9	51.9	8	6	1.6
Grovsnus White		45.3	49.9	53.2	55.7	10.4	7.9	4.6
Goteborgs Rape White		45.1	49	52.6	55.3	10.2	7.5	3.9
Kronan		43.2	45.3	49.5	51.1	7.9	6.3	2.1
Catch Licorice, mini		42.4	46.7	47.6	52.2	9.8	5.2	4.3
Catch White Licorice		46.5	49.6	53.2	55.9	9.4	6.7	3.1
Catch Dry White Eucalyptus, mini		22.4	29.2	24	27.5	5.1	1.6	6.8
Catch Dry White Licorice, mini		22.2	25.1	20.5	25.9	3.7	− 1.7	2.9
Granit White		39.1	40.8	42.3	44.7	5.6	3.2	1.7
Granit		43.2	49.9	50	53.7	10.5	6.8	6.7
Tre-Ankare White		46.4	51.7	52.9	56	9.6	6.5	5.3
Level		47.8	46.8	47.2	50	2.2	− 0.6	− 1
Skruf strong		36.3	48.7	48.7	52.3	16	12.4	12.4
LD original		44	48.2	47.5	51.6	7.6	3.5	4.2
Knox		40.6	45.4	47.1	49	8.4	6.5	4.8
Romeo y Julieta Habanos		45.9	47.7	51.3	52.5	6.6	5.4	1.8
Wise Citrus and Menthol (6 mg)		5.5	7.43	7.9	9.6	4.1	2.4	1.93
1847 original		36.8	43.8	45.9	47.5	10.7	9.1	7
Average P Snus		39.5	43.7	45.1	48.0	8.5	5.6	4.2
SD P Snus		10.2	10.3	11.9	11.7	3.17	3.30	2.77
General	L snus	46.4	49.9	57.2	57	10.6	10.8	3.5
Ettan		46.7	50.7	57.3	57.7	11	10.6	4
Grovsnus		46.4	50.5	56.9	57.7	11.3	10.5	4.1
Goteborgs rape		48.5	51.9	56.4	57.6	9.1	7.9	3.4
Kronan		48.5	50	58.1	57.3	8.8	9.6	1.5
Granit		44.8	49	54.5	54.3	9.5	9.7	4.2
LD original		48	50.9	48.5	55.8	7.8	0.5	2.9
Skruf strong		49	52.7	56.6	57.2	8.2	7.6	3.7
Knox		43.4	51.9	57.3	56.6	13.2	13.9	8.5
T. Montecristo		49.7	50	56	54.1	4.4	6.3	0.3
Average L Snus		47.1	50.8	55.9	56.5	9.4	8.74	3.61
SD L Snus		1.98	1.13	2.77	1.36	2.39	3.57	2.12

**Table 4:** Water and moisture contents for US STPs (CT, DS and HP)

	Style	Water (%)		Moisture (%)		BAT oven-KF (%)	Labstat oven—KF (%)	NIR—KF (%)
		Karl Fischer water	NIR water	Labstat oven moisture	BAT oven moisture			
Beech Nut	CT	21.3	24.1	18.7	27.6	6.3	− 2.6	2.8
Chattanooga		18.7	21.1	24.8	24.3	5.6	6.1	2.4
Durango		20.1	23.4	22.7	25.9	5.8	2.6	3.3
Lancaster		20.2	22.3	24.3	25.6	5.4	4.1	2.1
Levi Garrett		17.5	22.9	23.2	23.4	5.9	5.7	5.4
Morgans		18.8	22	22.8	24	5.2	4	3.2
Red man gold		21.1	25.8	26.4	27	5.9	5.3	4.7
Red man regular		20.6	25.3	25.8	27	6.4	5.2	4.7
Southern pride		21.2	24.5	25.6	26.7	5.5	4.4	3.3
Starr		18	22.9	24.8	26.1	8.1	6.8	4.9
Stoker 707 wintergreen		18.7	20.7	21.8	23.8	5.1	3.1	2
Taylors Pride		16	22.1	23.5	24	8	7.5	6.1
Trophy		19.2	23	24.1	24.9	5.7	4.9	3.8
Average CT		19.3	23.1	23.7	25.4	6.1	4.4	3.7
SD CT		1.60	1.52	2.02	1.43	0.96	2.51	1.31
Bruton	DS	5.8	7.12	9.37	9.2	3.4	3.57	1.32
Dental Sweet		4.5	7.93	10	9.5	5	5.5	3.43
Garrett		4.8	8.22	10	9	4.2	5.2	3.42
Honest		4.3	7.25	10	8.7	4.4	5.7	2.95
Square		7.4	7.37	8.52	8.6	1.2	1.12	− 0.03
Average DS		5.4	7.6	9.6	9.0	3.6	4.2	2.2
SD DS		1.28	0.47	0.65	0.37	0.48	1.92	1.53
Ariva Java	HP	3.1	1.82	3.53	3.8	0.7	0.43	− 1.28
Stonewall wintergreen		2.7	2.01	4.35	4.9	2.2	1.65	− 0.69
Average HP		2.9	1.92	3.94	4.35	1.45	1.04	− 1.0
SD HP		0.28	0.13	0.58	0.78	1.06	0.86	0.42

**Table 5:** Water and moisture contents for US STPs (SP, MS and plug)

	Style	Water (%)		Moisture (%)		BAT oven-KF (%)	Labstat oven—KF (%)	NIR—KF (%)
		Karl Fischer water	NIR water	Labstat Oven moisture	BAT Oven moisture			
Oliver twist original	SP	19.7	13.4	17.3	18.9	− 0.8	− 2.4	− 6.3
Copenhagen LC	MS	47.1	52.1	53.6	54.7	7.6	6.5	5
Copenhagen straight LC		50.1	53.1	54.3	54.6	4.5	4.2	3
Grizzly natural LC		49.6	53	54.8	55.3	5.7	5.2	3.4
Husky natural FC		51.4	53.9	55.7	56.1	4.7	4.3	2.5
Husky straight LC		51	54.5	56.2	56.9	5.9	5.2	3.5
Husky wintergreen		50.3	52.2	54.9	55.8	5.5	4.6	1.9
Kayak straight LC		50.4	50.8	54.4	53.3	2.9	4	0.4
Kodiak straight LC		48.8	51.7	53.9	54.3	5.5	5.1	2.9
Kodiak wintergreen		48	49.2	52.3	52.8	4.8	4.3	1.2
Silver creek		49.5	48.8	51.5	53.2	3.7	2	− 0.7
Skoal straight		50.3	52.7	54.9	55.4	5.1	4.6	2.4
Timber wolf natural FC		47.8	48.7	50	51.2	3.4	2.2	0.9
Timber Wolf Straight LC		50	51.2	55.7	55.6	5.6	5.7	1.2
Marlboro original LC		50.8	52.1	55.3	55.5	4.7	4.5	1.3
Red Seal natural FC		49.2	52.4	54.1	55.2	6	4.9	3.2
Red Seal natural LC		50.1	54.1	55.5	56.5	6.4	5.4	4
Average MS		49.6	51.9	54.2	54.8	5.1	4.5	2.3
SD MS		1.20	1.79	1.67	1.50	1.18	1.15	1.48
Cannonball	Plug	15.4	16.9	19.3	21.2	5.8	3.9	1.5

#### Sugar contents

**Fig. 3:**
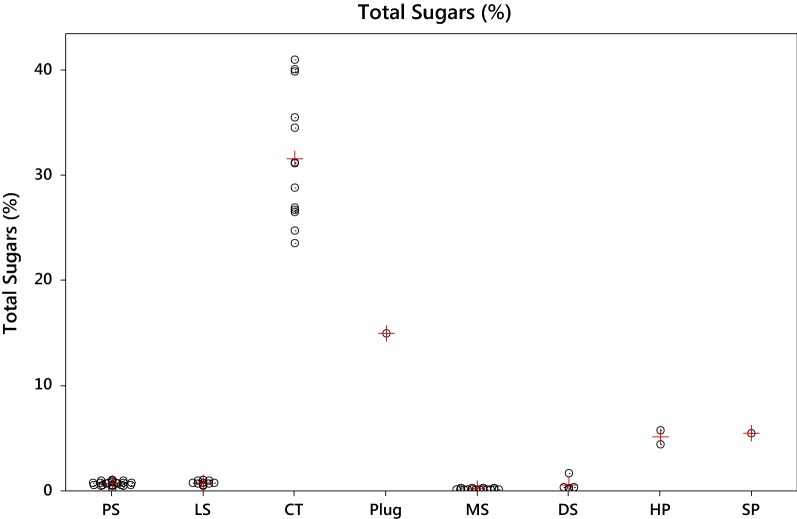
Individual (o) and average (+) values for total sugars (%) by product style

Individual and average levels of total sugars are shown by product style in Fig. 3. Total sugar levels were below 1% for L and P snus, DS and MS. Slightly higher levels (around 5%) were found in HP and SP. The plug product had 14.9% sugars. All the CT products had large sugar contents (average 32%, range 23–41%). The sugar levels in the CT and plug products exceed the levels naturally found in most cured tobaccos [41], due to added sugar in these products. Some small inconsistencies were observed in the relative magnitudes of reducing and total sugars for P Snus, L snus, MS, and DS samples, due to their sugar contents being close to the analytical LOQ.

#### Humectants

The data in Table 1 and Additional file 1: Tables S1–S8, show that humectants were widely used in Swedish L and P snus products, chewing tobacco and plug, but rarely in the other STPs examined in this study. They were used at the highest levels in L Snus (mean combined glycerol and propylene glycol contents of 6.5%), and at lower levels in CT (3.5%), P Snus (2.7%) and Plug (2.3%). Average values for the other STP categories were 0.5% with MS, 0.1% for DS and SP, and 0% for HP. Amongst the humectants there were differences in use of glycerol and PG. All the L Snus brands, the plug brand and all except 1 of the CT brands had glycerol levels between 1.4 and 6.4%. There were significantly higher levels of glycerol in the L Snus compared with the P Snus (3.0% vs 0.05%), potentially due to manufacturing factors. Most of the MS products also had no glycerol except for Kayak Straight LC (4.4%) and Silver Creek (3.2%). None of the HP, DS, SP and MS products contained significant levels of propylene glycol. CT and plug products contained up to 0.8%. Most of the P and L Snus products had levels of propylene glycol in the range: 1.9–3.9%. Three brands of P Snus had no propylene glycol: Catch Dry White Eucalyptus Mini, Catch Dry White Licorice Mini and Wise Citrus and Menthol. These were also the brands with the lowest moisture levels.

#### Sodium and chloride ions

**Fig. 4:**
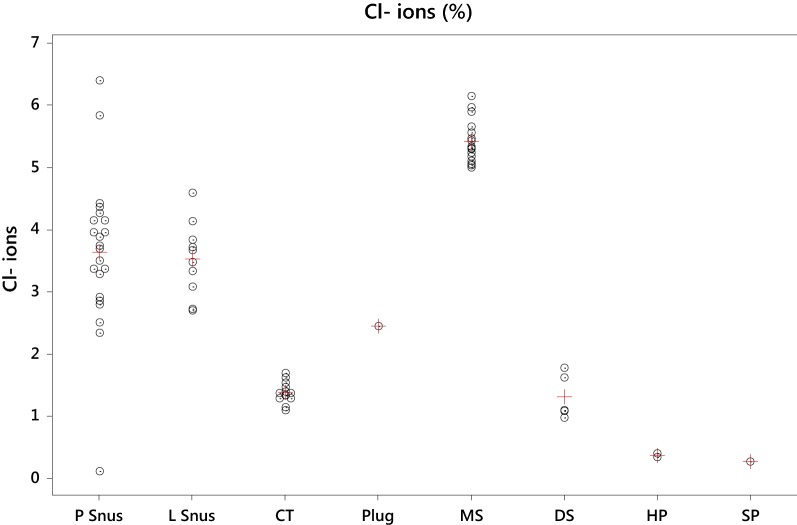
Individual (o) and average (+) values for chloride ions (%) by product style

The individual and average concentrations of chloride ions show significant differences between STPs (Fig. 4). HP and SP have low Cl^−^ concentrations (< 0.4%). CT and DS have Cl^−^ concentrations averaging 1.4%. Except for Wise Citrus and Menthol which contains no significant levels of Cl^−^, L and P snus products contain between 2.3 and 6.4% Cl^−^ averaging about 3.5%. The highest levels of Cl^−^ were found in the MS products all of which had levels of 5% and above.

The individual and average concentrations of sodium ions showed similar trends (Table 1 and Additional file 1: Tables S1–S8). CT, DS, HP and SP all have less than 1% Na^+^. L and P Snus products have much higher levels of Na^+^—between 1.8 and 3.5% for the high moisture brands and higher (4–4.2%) for some lower moisture brands. The MS products also have high Na^+^ concentrations averaging about 3%.

The natural Cl^−^ content of tobacco depends on soil Cl^−^ concentrations but typical DWB concentrations of 0.26% (Maryland), 0.69% (aromatic), 0.84% (flue-cured) and 0.91% (burley) have been reported [42]. Wyttenbach et al. [43] reported Cl^−^ levels between 0.36 and 1.64% DWB in a series of 20 different raw tobaccos. In the same study concentrations of sodium were in the range 0.015–0.09%.

The levels of Na^+^ and Cl^−^ in the current study were strongly correlated (R^2^ = 0.82), and the gradient of the linear regression between molar % contents was close (0.92) to unity, with an intercept pointing to a small excess of Cl^−^ over Na^+^. In addition, the ratios of Cl^−^ to Na^+^ for all the snus products (1.18–2.23) (except for Wise Citrus and Menthol (0.05)) and the MS products are consistent with significant quantities of sodium chloride (ratio 1.54) being added to these brands. Swedish Match adds sodium chloride (1.3–3.7% to its P snus brands and 3.7–4.5% to its L snus brands) as a flavour and preservative [39]. The relative concentration of Na^+^ and Cl^−^ in Wise Citrus and Menthol and in the snus brands with higher Na^+^ is consistent with addition of a sodium salt other than Cl^−^ such as sodium carbonate or bicarbonate to these products, as noted previously.

The results here indicate that significant quantities of Na^+^ and Cl^−^ salts have been added to the L and P snus (except for Cl^−^ in Wise Citrus and Menthol) and MS products, and smaller quantities to the CT, DS and Plug products.

#### Nicotine and pH

Individual and average levels of total nicotine are given in the Additional file 1: Tables S1–S8, and Table 1. Average nicotine concentrations were highest for the single SP product (29.5 mg/g) and then in order of decreasing levels: DS (20.6 mg/g), MS (12.2 mg/g), the plug product (10.9 mg/g), P Snus (9.9 mg/g), HP (8.4 mg/g), L Snus (7.9 mg/g), and CT (6.7 mg/g). The variation in nicotine among the brands was fairly similar for each style except for P Snus where the 3 lowest moisture brands (Catch Dry White Eucalyptus Mini, Catch Dry White Licorice Mini and Wise Citrus and Menthol) had proportionately higher nicotine than the other P Snus products and contributed to the higher variation in nicotine in this category.

pH also varied significantly between styles of STP. The L and P snus products were basic, and had the highest pHs (average 8.5, range 7.5–9.4), with no differences on average between the loose and pouch products; the two HP products (7.9–8.1) and MS (7.8, range 6.4–8.4), had similar albeit slightly lower average pH values. The other STP categories had lower average pH values, and other than one DS product (Bruton) were not basic: DS (6.4, range 5.9–7.2), CT (6.2, range 5.6–6.5), plug and SP (5.3). Many of the basic STPs appear to have ammonium, sodium or potassium carbonates added to the tobacco [39, 44–46]. Carbonates are also added to Bruton DS [44], which has the highest pH of this STP category. The range of values measured with L and P snus products and MS were wider than found with the other STP classes, may reflect differences in additive (e.g. carbonate) level or processing methods between different products and manufacturers.

**Fig. 5:**
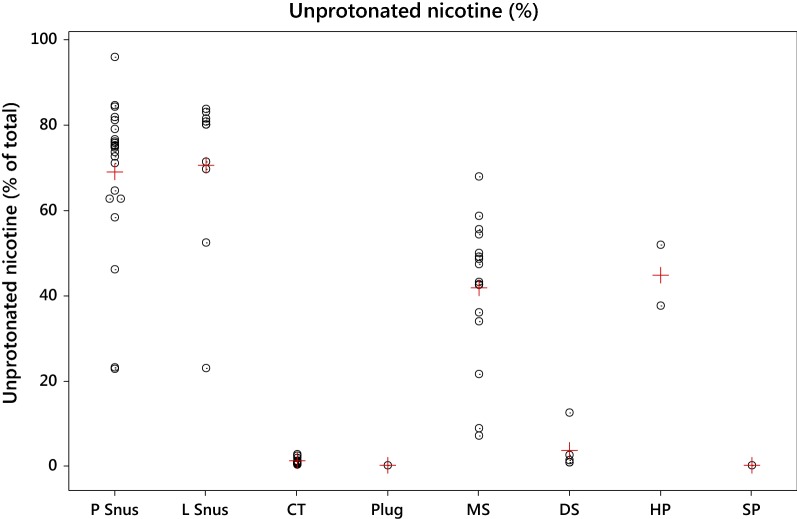
Individual (o) and average (+) values for  % unprotonated nicotine (of total nicotine content) by product style

The pH values were used to estimate the levels of unprotonated (free) nicotine in the products using the Henderson-Hasselbalch approximation [47]. These are shown by product style in Fig. 5. The higher pH of the L and P snus products ensures that most of the nicotine in these products is in the unprotonated form; levels in MS are slightly lower. The much lower pH of the DS, CT, SP and plug products keeps most of the nicotine in the protonated form. The SP product for example has three times more total nicotine than the average snus product, but ten times less unprotonated nicotine.

#### Ash and tobacco contents

In Fig. 1 the quantity called “balance” consists of tobacco (minus nicotine) and any other ingredients that were not measured (such as flavours). It is clear from Fig. 1 that with the Swedish L and P snus products, MS and CT, tobacco is a minority constituent. For these four STP types tobacco comprises less than 40% of the product mass, with water, sugars (CT), fleece (P snus), sodium and chloride and humectants making up around 60–70% of the product masses. In contrast tobacco makes up the majority of the product masses with plug (> 55%), SP (> 70%) and particularly DS (> 85%). These findings are surprising but show the heterogeneity of product composition amongst contemporary STPs.

The ash values in Table 1 and Additional file 1: Tables S1–S8, are derived from inorganic material naturally present in the tobacco plus components such as sodium chloride, which is added. Within the L and P snus products (with the exception of Wise Citrus and Menthol) there was a good correlation between ash and the sum of Na^+^ and Cl^−^ concentrations (R^2^ = 0.762). The CT products have both low Na^+^ and Cl^−^ concentrations and low ash, but within this product group their levels were not correlated. DS has low Na^+^ and Cl^−^ but high ash levels. This may be explained in part by the use in DS of high levels of stem [48] which produces greater quantities of ash than lamina [49], and also the low water content of DS.

### Comparison of moisture and water measurement methods

In the present study moisture contents were measured by two variations of the oven method, while water content was measured by Karl Fischer coulometric titration and NIR reflectance spectroscopy. The Karl Fischer approach was regarded as the reference method for this study, given its robust specificity and long-term acceptance for water determination. The aim of this study was to establish the degree of consistency or level of discrepancies provided by the different measurement techniques with contemporary STPs. It has been established previously that there are drawbacks to all methods regarding tobacco measurements. For example, with the Karl Fischer titration method the reagent used also reacts with low molecular weight carbonyls known to be present in STPs [6], potentially leading to elevated readings, and the solvent extraction procedure may not remove all the water from the sample [50] potentially leading to depressed values. With the NIR reflectance method significant variation in substrate type from sample to sample may lead to measurement errors. In addition, use of another reference water measurement technique, often Karl Fischer titration, is required for calibration. The major criticism with the oven method is that volatiles are determined in addition to water, potentially leading to a higher value than water-specific analyses. Below 75 °C only water is removed from uncased tobacco while above 75 °C there is a loss of volatiles and decomposition starts [51]. The oven drying method is based on the observation that, provided the tobacco sample is heated for less than 4 h, at temperatures between 75 and 100 °C, the rate of loss of water is much greater than the loss of volatiles and decomposition. However, tobaccos which contain volatile casings or humectants will register greater weight losses, and hence moistures, than tobaccos without such ingredients. Bourlas et al. [52] demonstrated that the volatile ingredients of casings applied to tobaccos influence moisture determinations. They found that for a series of cased tobaccos the oven method gave, on average, 2.4% higher moisture levels than found using Karl Fischer titration. This compared with uncased tobaccos for which the oven method gave on average 1.2% higher moisture levels than with the Karl Fischer method. Ryan and Parrish [53] analysed the volatiles that were generated during oven moisture drying of cased tobaccos at 100 °C. Analysis of the samples pre- and post- oven heating indicated average losses of 52% of the propylene glycol, 8% of the glycerol, 48% of the soluble ammonia, 8% of the total alkaloids, and 31% of the acetic acid initially present. Lewis [54] found that oven drying at 100 °C for 3 h removed 26–100% of propylene glycol from tobacco and up to 55% of the glycerol. The amounts of propylene glycol and glycerol lost were found to be strongly dependent on the substrate. Bourlas et al. [52] found that at 100 °C significant decomposition of the reducing sugars (glucose and fructose) can occur, which also contributed to the weight loss. The current study examined the impact of these factors on moisture and water measurement from contemporary STPs.

**Fig. 6:**
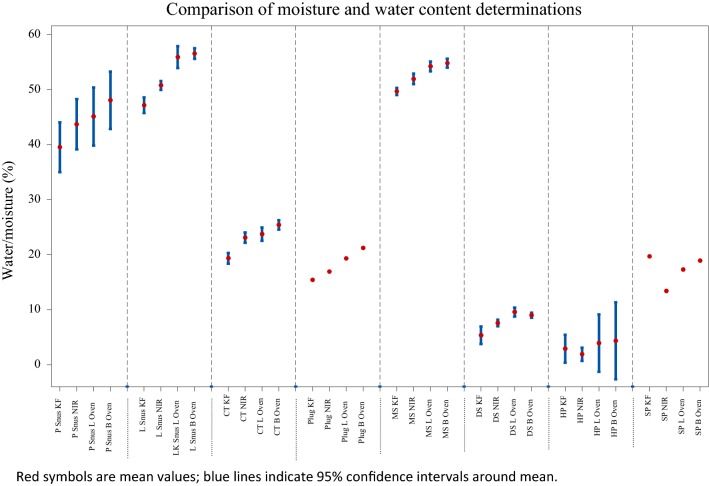
Differences in water and moisture content by style and by analytical method

The moisture and water content results obtained for the STPs by the different methods are shown in Tables 3, 4 and 5, as well as the average values by style of STP, which is illustrated in Fig. 6. With a few exceptions the measured values were lowest using the Karl Fischer method, highest using the BAT oven method, and intermediate using NIR and the Labstat oven method. The NIR method gives, on average, lower values than either oven method, probably reflecting the fact that unlike the oven method NIR is not sensitive to volatiles other than water in the sample.

Exceptions to these trends were found with CatchDry White Eucalyptus mini P snus where BAT oven moisture < NIR water, Square dry snuff where Karl Fischer water = NIR water and Silver Creek moist snuff where Karl Fischer water > NIR water. For the HP and SP products, Karl Fischer water > NIR water and for the SP product, Karl Fischer water > BAT and Labstat oven moisture and NIR water.

Pearson correlations (R) between moistures and water contents measured by the 4 methods are shown in Table 6. All the correlations were significant at p = 0.000. There was a slightly better correlation between BAT oven moisture and NIR water (R = 0.996) than between NIR water and Karl Fischer water (R = 0.988) and between BAT oven moisture and Karl Fischer water (R = 0.987). There was a good correlation between BAT and Labstat oven moistures (R = 0.994). On average the Labstat oven moisture method gave lower moisture values than the BAT method for all the styles of STPs except dry snuff; however, the Labstat oven moisture method still gave consistently higher values than the Karl Fischer water analysis. The higher temperature used in the BAT method would probably result in more volatiles being driven from the sample, resulting in higher moisture values.

**Table 6:** Pearson correlations (R) between moisture and water content methods

	Labstat oven (moisture)	KF (water)	NIR (water)
BAT oven (moisture)	0.994	0.987	0.996
Labstat oven (moisture)		0.986	0.991
KF (water)			0.988

All p-values were 0.000

The differences between moisture values determined by the two oven methods and the water contents by NIR and Karl Fischer analysis depended in part on the style of the STP (Fig. 6). Differences between BAT oven moistures and Karl Fischer water contents were greatest with both L and P Snus products (9%), CT and MS were 5–6% higher from the BAT oven method, and differences were smallest with the hard and soft pellet products (around 1%), with the latter oven moistures lower than the Karl Fischer water. Recognizing that the differences between the BAT oven moistures and the Karl Fischer water may be explained in part by losses of volatile or heat-sensitive ingredients, this parameter was used to determine if these differences correlated with any particular STP ingredient. The differences between BAT oven moistures and Karl Fischer water values were calculated, and correlations determined between these differences and levels of glycerol, propylene glycol, total sugars, reducing sugars and nicotine (Table 7). Propylene glycol (BP 188 °C) showed a correlation (R = 0.72, p < 0.001) with the difference between BAT oven moistures and Karl Fischer water. There were no significant correlations with levels of the less volatile (BP 290 °C) glycerol (p = 0.484) or with reducing sugars (p = 0.371) and total sugars (p = 0.327). The latter suggests that sugar decomposition during oven drying does not make a major contribution to tobacco weight loss under these conditions. The differences between BAT oven moistures and Karl Fischer waters were also significantly (p < 0.05) and negatively correlated with ammonia [12] (R = − 0.39), nicotine (R = − 0.48) and ash (R = − 0.395).

**Table 7:** Pearson correlations (R) and significance (p) between ingredients and difference between oven moistures and KF water

	Glycerol	Propylene Glycol	Total Sugars	Reducing Sugars	Ammonia*	Nicotine	Ash
Slope	0.152	1.40	− 0.029	− 0.056	− 0.016	− 0.300	− 0.341
R	0.085	0.72	− 0.119	− 0.109	− 0.39	− 0.48	− 0.395
p	0.484	< 0.001	0.327	0.371	< 0.001	< 0.001	< 0.001

* Taken from McAdam et al. [12]

There were also consistently higher values from the NIR method than from the Karl Fischer approach. The differences were smaller than seen with both oven methods but were present with most STP categories. With P Snus NIR gave on average higher values than Karl Fischer by 4–4.5%, with L Snus and CT the difference was 3.6–3.7%, DS and MS gave differences of 2.2–2.3%. In contrast HP and SP gave higher values by Karl Fischer than NIR (1–6%). Although these discrepancies are lower than those found with the oven methods they still show divergence from the water values determined by the reference Karl Fischer method. The differences between NIR and Karl Fischer methods are greatest among those categories containing highest humectant levels. The NIR analysis approach relies upon determination of the intensity of a combined O–H bond stretching and H–OH bending band in the IR spectrum. The presence of OH groups in glycerol and propylene glycol may interfere with the OH stretching band, and matrix-matched calibrations may be required to improve performance of the NIR technique across a range of STPs.

The WHO TobReg study group [22] did not specify the moisture method to be used for conversion of actual toxicant contents to DWB values. If oven methods are used then it can be expected that STPs with higher levels of humectant, particularly propylene glycol, will register higher moisture values than the actual water content due to losses of volatiles. This in turn will lead to higher DWB concentrations being calculated. Using the data obtained in this study across all STP categories and comparing the highest oven method with Karl Fischer measurements suggests an average error of 10–15% would arise. A similar, albeit smaller, effect would be observed with the NIR approach.

It is also notable that the conversion of wet weight actual contents to dry weight values will have differing impact on products that differ in water content, with greater effect on wetter STPs. On average the concentrations of the compounds in the moister STPs (MS & L and P snus) would approximately double when normalised to a DWB. In contrast there are much smaller increases in concentrations in the drier STPs (DS, CT and pellet products) when the results are normalised to a DWB; in the case of DS the adjustment would be an increase of around 5%. Even within styles of STP, normalisation to DWB can have a significant impact. Using nicotine as an example of a tobacco constituent, the actual concentration in Catch Dry White Licorice Mini (16.95 mg/g) as used by consumers is 50% higher than in Romeo y Julieta Habanos (11.3 mg/g). However, due to differences in their water contents (22.2% and 45.9% respectively) after normalisation to DWB, the two products appear to have similar concentrations of nicotine. Hence conversion to DWB can misrepresent actual concentrations in products to a degree that increases with product water content.

## Conclusions

In this study we have quantified the major constituents of 70 STPs sold in the US and Sweden comprising moist and dry snuffs, chewing tobaccos and plug, hard and soft pellet products, loose and portion snus. Reducing and total sugars, ash, glycerol, propylene glycol, sodium and chloride ions, nicotine and moisture/water were analysed. Each of the added ingredients, including water, plays a specific role in ensuring the acceptability and integrity of the product. The levels of most of the components varied by at least an order of magnitude across different styles of product. For example, Karl Fischer water contents ranged from an average of 2.9% for the HP products to 47.1% on average for L snus; Na and Cl ions from 0.04% and 0.37% respectively in HP products to 3.2% and 5.4% in MS, and total sugars from 0.1% in MS to 31.6% in CT. Our results show that STPs are composite materials that differ greatly in composition, and in which tobacco is often a variable component (30–90%).

Comparison of four commonly used approaches for tobacco moisture and water determination showed that two different oven moisture methods and an NIR water approach gave systematically higher values than Karl Fischer water measurements with these STPs. The greatest discrepancies were obtained with oven techniques. There is vast diversity in the water/moisture contents of different styles of STPs and normalising chemical contents of STPs to a dry weight basis can misrepresent actual concentrations in products to a degree that increases with product water/moisture content.

## Additional file

**Additional file 1.** Individual brand-by-brand constituent data. **Table S1.** Constituent data for Swedish Portion Snus brands. **Table S2.** Constituent data for Swedish Loose Snus brands. **Table S3.** Constituent data for US Moist Snuff brands. **Table S4.** Constituent data for US Chewing Tobacco brands. **Table S5.** Constituent data for the US Plug brand. **Table S6.** Constituent data for US Dry Snuff brands. **Table S7.** Constituent data for US Hard Pellet brands. **Table S8.** Constituent data for the US Soft Pellet brand.

## Abbreviations

B[a]P: benzo[a]pyrene
BAT: British American Tobacco
BLD: below the limit of detection
CORESTA: Cooperation Centre for Scientific Research Relative to Tobacco
CT: chewing tobacco
DS: dry snuff
DWB: dry weight basis values; the value after correction of measured contents for moisture content
FDA: US Food and Drug Administration
HP: hard pellet
HPHC: Harmful and Potentially Harmful Constituents
IARC: International Agency for Research on Cancer
L Snus: loose snus
ISO: International Organization for Standardization
MS: moist snuff
NQ: not quantified
PAH: polycyclic aromatic hydrocarbon
P Snus: portion snus
SP: soft pellet
STP: smokeless tobacco product
TPSAC: FDA Tobacco Product Scientific Advisory Committee
TSNA: tobacco specific nitrosamines
WWB: wet weight basis values; the value as measured for the STP in its “as sold” form
