# Supplementary material for: The composition of contemporary American and Swedish smokeless tobacco products
Source: BMC Chem. 2019 Mar 19;13(1):31. doi: 10.1186/s13065-019-0548-0 (PMC6661804; doi:10.1186/s13065-019-0548-0)
Supplement: Supplementary file 1 — Additional file 1. Individual brand-by-brand constituent data. Table S1. Constituent data for Swedish Portion Snus brands. Table S2. Constituent data for Swedish Loose Snus brands. Table S3. Constituent data for US Moist Snuff brands. Table S4. Constituent data for US Chewing Tobacco brands. Table S5. Constituent data for the US Plug brand. Table S6. Constituent data for US Dry Snuff brands. Table S7. Constituent data for US Hard Pellet brands. Table S8. Constituent data for the US Soft Pellet brand. [file 13065_2019_548_MOESM1_ESM.pdf]

Supplementary Table 1: Constituent data for Swedish Portion Snus brands

Product	STP Style	Red Sugars (%)		Total Sugars (%)		Ash (%)		Glycerol (%)		PG (%)		Na^+ (%)		Cl^- (%)		Nicotine (mg/g)		pH	
		Mean	SD	Mean	SD	Mean	SD	Mean	SD	Mean	SD	Mean	SD	Mean	SD	Mean	SD	Mean	SD
General White		0.7	0.07	0.6	0.07	15.2	0.04	0.04	0.00	3.31	0.01	2.68	0.07	3.74	0.08	7.85	0.07	8.8	0.03
General, Mini		0.6	0.00	0.5	0.07	14.5	0.08	0.04	0.00	3.72	0.01	2.31	0.02	2.85	0.04	7.80	0.28	8.3	0.02
General		0.9	0.00	0.8	0.00	15.2	0.12	0.07	0.00	3.30	0.00	2.73	0.03	3.96	0.04	8.00	0.14	8.5	0.01
Ettan		0.6	0.07	0.5	0.07	14.6	0.04	0.06	0.00	3.67	0.04	2.35	0.06	2.34	0.02	8.10	0.14	8.5	0.01
Grovsnus		0.7	0.00	0.7	0.00	14.6	0.12	0.06	0.00	3.55	0.00	2.26	0.12	2.80	0.15	8.20	0.28	8.6	0.01
Grovsnus White		0.9	0.00	0.9	0.00	14.7	0.01	0.06	0.00	3.42	0.00	2.73	0.03	3.69	0.03	8.20	0.00	8.6	0.01
Goteborgs Rape White		0.8	0.00	0.7	0.00	15	0.02	0.05	0.00	3.33	0.00	2.72	0.06	3.88	0.05	8.20	0.00	8.5	0.01
Kronan		1	0.00	1	0.00	15	0.01	0.04	0.00	3.49	0.00	2.45	0.02	3.29	0.06	8.70	0.14	8.2	0.02
Catch Licorice Mini		0.8	0.00	0.7	0.07	14.2	0.07	0.04	0.00	3.81	0.03	2.29	0.04	3.37	0.04	7.90	0.14	8.5	0.00
Catch White Licorice		0.6	0.00	0.5	0.00	14.6	0.03	0.04	0.00	3.22	0.00	2.59	0.02	4.36	0.05	8.00	0.00	8.6	0.00
Catch Dry White Euc Mini		0.8	0.07	0.7	0.07	23.2	0.08	0.11	0.00	0.00	0.00	4.02	0.11	5.83	0.08	16.95	0.07	7.5	0.02
Catch Dry White Lic Mini	P Snus	0.8	0.00	0.7	0.00	23.8	0.01	0.12	0.00	0.00	0.00	4.21	0.16	6.40	0.32	16.95	0.35	7.6	0.05
Granit White		0.9	0.00	0.9	0.00	18.7	1.09	0.06	0.00	3.95	0.00	2.65	0.71	4.42	1.17	9.45	0.35	8.3	0.01
Granit		0.5	0.07	0.4	0.00	15.1	0.01	0.04	0.00	3.88	0.00	2.30	0.25	3.50	0.32	7.55	0.07	8.6	0.01
Tre Ankare White		0.7	0.07	0.5	0.00	14.7	0.19	0.04	0.00	3.24	0.01	2.66	0.08	4.27	0.15	7.70	0.14	8.7	0.00
LD Original		0.5	0.00	0.4	0.00	15.9	0.11	0.00	0.00	2.74	0.00	1.79	0.04	3.96	0.04	6.65	0.07	8.7	0.02
Level		0.6	0.00	0.6	0.00	16.4	0.08	0.00	0.00	2.93	0.01	1.86	0.02	4.15	0.03	6.65	0.21	8.5	0.01
Skruf Strong		0.8	0.00	0.7	0.00	13.7	0.04	0.06	0.00	2.94	0.01	2.04	0.01	2.91	0.01	10.70	0.00	8.8	0.01
Knox		0.9	0.00	0.9	0.07	13.8	0.02	0.06	0.00	2.95	0.01	1.70	0.01	2.51	0.02	10.60	0.14	8.0	0.01
Wise Citrus & Menthol		0.4	0.10	0.2	0.08	23.9	2.05	0.17	0.01	0.00	0.00	2.43	0.42	0.12	0.02	23.65	3.59	9.5	0.04
T. Romeo Y Julieta		0.5	0.07	0.4	0.07	14.3	0.01	0.00	0.00	3.48	0.01	2.07	0.03	3.37	0.02	11.30	0.00	8.3	0.00
1847 Original		0.4	0.00	0.5	0.00	17.8	0.08	0.00	0.00	3.48	0.01	3.53	0.02	4.15	0.07	8.10	0.00	8.6	0.00
Average/SD* P snus		0.70	0.17	0.63	0.20	16.31	3.20	0.05	0.04	2.93	1.23	2.56	0.64	3.63	1.24	9.87	4.14	8.46	0.41

* Indicates SD of collated individual brand measurements, i.e. product category SD

Values in red are product category means and SDs

Supplementary Table 2: Constituent data for Swedish Loose Snus brands

Product	STP Style	Red Sugars (%)		Total Sugars (%)		Ash (%)		Glycerol (%)		PG (%)		Na^+ (%)		Cl^- (%)		Nicotine (mg/g)		pH	
		Mean	SD	Mean	SD	Mean	SD	Mean	SD	Mean	SD	Mean	SD	Mean	SD	Mean	SD	Mean	SD
General Loose	L Snus	0.8	0.00	0.7	0.00	14.1	0.20	2.95	0.02	3.55	0.01	2.76	0.04	3.67	0.08	7.10	0.14	8.7	0.00
Ettan Loose		0.8	0.00	0.7	0.00	14.1	0.05	2.91	0.00	3.48	0.01	2.72	0.03	3.72	0.03	7.30	0.00	8.8	0.00
Grovsnus Loose		0.9	0.00	0.9	0.00	13.3	0.11	3.66	0.01	3.60	0.00	2.56	0.03	3.33	0.10	7.30	0.00	8.7	0.00
Goteborgs Rape Loose		1	0.00	0.9	0.07	13.9	0.05	3.59	0.01	3.49	0.01	2.21	0.83	3.08	1.18	6.60	0.14	8.4	0.01
Kronan Loose		1	0.00	1	0.07	13.3	0.01	3.64	0.03	3.56	0.02	2.49	0.04	3.48	0.05	7.65	0.07	8.8	0.01
Granit Loose		0.6	0.00	0.6	0.07	13.9	0.12	4.71	0.03	3.39	0.01	2.41	0.05	4.13	0.01	7.60	0.14	8.5	0.01
LD Loose		0.8	0.00	0.7	0.00	15.6	0.01	1.39	0.00	2.35	0.00	2.11	0.05	4.59	0.02	7.05	0.07	8.7	0.00
Skruf Strong Loose		0.6	0.00	0.5	0.00	12.1	0.04	2.42	0.01	2.63	0.01	1.85	0.01	2.70	0.02	9.90	0.14	8.1	0.02
T. Montecristo Habanos Loose		0.4	0.00	0.4	0.07	13.4	0.15	6.39	0.05	1.90	0.00	2.05	0.04	3.83	0.08	8.95	0.07	7.5	0.07
Knox Loose		0.4	0.00	0.6	0.00	12.6	0.13	2.64	0.02	2.28	0.01	2.01	0.02	2.72	0.05	9.80	0.00	8.7	0.02
Average/SD* L snus		0.73	0.22	0.70	0.19	13.63	0.95	3.43	1.37	3.02	0.66	2.32	0.32	3.53	0.60	7.93	1.18	8.49	0.39

* Indicates SD of collated individual brand measurements, i.e. product category SD

Values in red are product category means and SDs

Supplementary Table 3: Constituent data for US Moist Snuff brands

Product	STP Style	Red. Sugars (%)		Total Sugars (%)		Ash (%)		Glycerol (%)		PG (%)		Na^+ (%)		Cl^- (%)		Nicotine (mg/g)		pH	
		Mean	SD	Mean	SD	Mean	SD	Mean	SD	Mean	SD	Mean	SD	Mean	SD	Mean	SD	Mean	SD
Copenhagen LC		0.1	-	0.1	-	15.7	0.02	0	0	0	0	2.8	0.03	5.56	0.07	10.8	-	8.1	0.03
Copenhagen Straight LC		0.3	-	0.1	-	16.5	0.07	0	0	0	0	2.84	0.01	5.33	0.01	8.64	-	8.2	0.01
Grizzly Natural LC		0.2	-	0.1	-	17	0.03	0	0	0	0	3.18	0	5.89	0.04	15.9	-	8.1	0.02
Husky Natural FC		0.1	-	0.1	-	15.7	0.19	0	0	0	0	2.61	0.05	5.46	0.02	12.6	-	8.0	0.01
Husky Straight LC		0.2	-	0.1	-	15.9	0.07	0	0	0	0	2.82	0.04	5.03	0.06	11.6	-	8.1	0.01
Husky Wintergreen		0.1	-	0.1	-	16.3	0.18	0	0	0	0	3.07	0.08	5.23	0.01	12.9	-	7.5	0.03
Kayak Straight LC		0.2	-	0.2	-	16.5	0.07	4.36	0.01	0	0	3.28	0.12	5.43	0.03	10.9	-	7.1	0.02
Kodiak Straight LC	MS	0.2	-	0.1	-	18.1	0.08	0	0	0	0	3.18	0.12	6.14	0.08	11.2	-	8.2	0.02
Kodiak Wintergreen		0.3	-	0.2	-	18.1	0.04	0	0	0	0	3.47	0.05	5.96	0.05	10.8	-	8.4	0.01
Silver Creek		0.1	-	0.1	-	17.2	0.02	3.18	0.02	0.02	0.03	3.26	0.11	5.18	0.09	11.5	-	7.0	0.03
Skoal Straight		0.1	-	0.1	-	16.4	0.18	0	0	0	0	3.19	0.01	5.31	0.02	13.6	-	7.8	0.01
Timberwolf Natural FC		0.1	-	0.1	-	16.9	0.05	0	0	0.05	0	3.55	0.04	5.3	0.18	13.1	-	8.0	0.00
Timberwolf Straight LC		0.2	-	0.2	-	15.8	0.04	0	0	0	0	3.01	0.01	4.99	0.12	13.3	-	8.0	0.01
Marlboro Original LC		0.1	-	0.1	-	17.7	0.04	0	0	0	0	4.15	0.03	5.65	0.13	12.6	-	7.9	0.01
Red Seal Natural FC		0.1	-	0.1	-	16.9	0.15	0	0	0	0	3.14	0.04	5.1	0.08	13	-	7.8	0.00
Red Seal Natural LC		0.3	-	0.2	-	15.7	0.12	0	0	0	0	2.79	0.02	5.05	0.09	12.9	-	7.9	0.02
Average/SD* MS		0.17	0.08	0.13	0.04	16.65	0.82	0.47	1.35	0.00	0.01	3.15	0.37	5.41	0.35	12.21	0.08	7.8	0.08

- indicates not reported

* Indicates SD of collated individual brand measurements, i.e. product category SD

Values in red are product category means and SDs

Supplementary Table 4: Constituent data for US Chewing Tobacco brands

Product	STP Style	Red. Sugars (%)		Total Sugars (%)		Ash (%)		Glycerol (%)		PG (%)		Na^+ (%)		Cl^- (%)		Nicotine (mg/g)		pH	
		Mean	SD	Mean	SD	Mean	SD	Mean	SD	Mean	SD	Mean	SD	Mean	SD	Mean	SD	Mean	SD
Beech Nut	CT	13.4	-	31.1	-	12.1	0.91	5.54	0.04	0.54	0.01	0.71	0.02	1.37	0.03	8.6	-	5.8	0.04
Chattanooga		25.4	-	41	-	13.0	1.34	3.65	0.04	0	0	0.62	0	1.37	0.02	7.9	-	5.9	0.02
Durango		13.4	-	34.5	-	11.6	1.72	2.95	0.07	0.84	0.02	0.73	0.02	1.29	0.15	4.7	-	6.0	0.02
Lancaster		24.3	-	40.1	-	9.86	1.13	2.81	0	0.33	0	0.58	0	1.14	0.01	8.1	-	6.0	0.02
Levi Garrett		11.2	-	23.5	-	13.3	1.57	2.75	0.01	0.48	0	0.77	0.01	1.62	0.14	6.7	-	6.5	0.16
Morgans		10.5	-	26.9	-	10.7	0.49	2.65	0.14	0.48	0.01	0.82	0.01	1.39	0	3.4	-	6.5	0.01
Redman Gold		8.5	-	31.2	-	8.67	0.09	2.95	0.02	0.5	0.02	0.62	0.02	1.47	0.03	7.4	-	6.1	0.07
Redman Regular		7.3	-	26.5	-	10.0	0.61	2.39	0.02	0.74	0.04	0.73	0.03	1.69	0.05	9.6	-	6.2	0.05
Southern Pride		9.8	-	28.8	-	14.0	6.95	3.1	0.04	0.46	0.01	0.75	0.02	1.54	0.01	6.9	-	6.1	0.01
Starr		24.3	-	39.9	-	9.28	0.28	2.56	0.02	0.42	0	0.6	0	1.29	0.03	8.2	-	5.6	0.03
Stoker 707 Wintergreen		13.3	-	24.7	-	10.6	0.21	0.28	0.04	0.17	0	0.71	0.01	1.34	0.06	4.7	-	5.8	0.04
Taylors Pride		8	-	26.7	-	11.0	0.05	4.55	0.08	0.07	0	0.79	0.01	1.33	0.01	5.7	-	6.3	0.08
Trophy		8.4	-	35.5	-	9.43	0.24	3.77	0.02	0.75	0	0.6	0.03	1.1	0.02	5.1	-	6.1	0.02
Average/SD* CT		13.7	6.60	31.6	6.10	11.0	1.66	3.1	1.22	0.4	0.25	0.7	0.08	1.4	0.17	6.7	1.84	6.1	0.27

\- indicates not reported

* Indicates SD of collated individual brand measurements, i.e. product category SD

Values in red are product category means and SDs

Supplementary Table 5: Constituent data for the US Plug brand

Product	STP Style	Red. Sugars (%)		Total Sugars (%)		Ash (%)		Glycerol (%)		PG (%)		Na^+ (%)		Cl^- (%)		Nicotine (mg/g)		pH	
		Mean	SD	Mean	SD	Mean	SD	Mean	SD	Mean	SD	Mean	SD	Mean	SD	Mean	SD	Mean	SD
Cannonball	Plug	12	-	14.9	-	12.8	0.18	1.69	0.12	0.62	0.01	1.46	0.03	2.45	0.03	10.9	-	5.3	0.13

- indicates not reported

Supplementary Table 6: Constituent data for US Dry Snuff brands

Product	STP Style	Red. Sugars (%)		Total Sugars (%)		Ash (%)		Glycerol (%)		PG (%)		Na^+ (%)		Cl^- (%)		Nicotine (mg/g)		pH	
		Mean	SD	Mean	SD	Mean	SD	Mean	SD	Mean	SD	Mean	SD	Mean	SD	Mean	SD	Mean	SD
Bruton	DS	0.2	-	0.1	-	24.2	0.05	0.04	0.06	0	0	0.92	0.01	1.62	0.19	18.5	-	7.2	0.01
Dental Sweet		1.4	-	1.6	-	20.7	0.05	0.2	0	0.04	0	0.39	0.01	1.78	0	17.2	-	6.0	0.01
Garrett		0.5	-	0.3	-	20.1	0.05	0.11	0	0	0	0.01	0	0.97	0.02	22.4	-	6.0	0.02
Honest		0.4	-	0.3	-	20.7	0.06	0.1	0	0	0	0.01	0	1.1	0.02	20.2	-	6.2	0.00
Square		0.3	-	0.2	-	21.8	0.07	0	0	0	0	0.04	0	1.08	0.02	24.9	-	6.5	0.00
Average/SD* DS		0.56	0.48	0.5	0.62	21.5	1.65	0.09	0.08	0.008	0.02	0.274	0.40	1.31	0.36	20.64	3.08	6.4	0.51

\- indicates not reported

* Indicates SD of collated individual brand measurements, i.e. product category SD

Values in red are product category means and SDs

Supplementary Table 7: Constituent data for US Hard Pellet brands

Product	STP Style	Red. Sugars (%)		Total Sugars (%)		Ash (%)		Glycerol (%)		PG (%)		Na^+ (%)		Cl^- (%)		Nicotine (mg/g)		pH	
		Mean	SD	Mean	SD	Mean	SD	Mean	SD	Mean	SD	Mean	SD	Mean	SD	Mean	SD	Mean	SD
Ariva Java	HP	3.6	-	4.4	-	22.6	0.00	0	0	0	0	0.04	0	0.4	0.21	6.8	-	8.1	0.01
Stonewall Winterg		5.1	-	5.7	-	20.7	0.05	0	0	0	0	0.04	0	0.34	0.07	10	-	7.9	0.00
Average/SD* HP		4.35	1.06	5.05	0.92	21.7	1.35	0	0	0	0	0.04	0	0.37	0.04	8.4	2.26	8.0	0.18

- indicates not reported

* Indicates SD of collated individual brand measurements, i.e. product category SD

Values in red are product category means and SDs

Supplementary Table 8: Constituent data for the US Soft Pellet brand

Product	STP Style	Red. Sugars (%)		Total Sugars (%)		Ash (%)		Glycerol (%)		PG (%)		Na^+ (%)		Cl^- (%)		Nicotine (mg/g)		pH	
		Mean	SD	Mean	SD	Mean	SD	Mean	SD	Mean	SD	Mean	SD	Mean	SD	Mean	SD	Mean	SD
Oliver Twist Original	SP	2.1	-	5.4	-	13.3	0.02	0.11	0	0	0	0.17	0	0.27	0.01	29.5	-	5.3	0.0

- indicates not reported
